# The Immunopeptidomics Ontology (ImPO)

**DOI:** 10.1093/database/baae014

**Published:** 2024-06-10

**Authors:** Daniel Faria, Patrícia Eugénio, Marta Contreiras Silva, Laura Balbi, Georges Bedran, Ashwin Adrian Kallor, Susana Nunes, Aleksander Palkowski, Michal Waleron, Javier A Alfaro, Catia Pesquita

**Affiliations:** INESC-ID, Instituto Superior Técnico, Universidade de Lisboa, Rua Alves Redol, 9, Lisboa 1000-029, Portugal; LASIGE, Faculdade de Ciências da Universidade de Lisboa, Campo Grande, Lisboa 1749-016, Portugal; LASIGE, Faculdade de Ciências da Universidade de Lisboa, Campo Grande, Lisboa 1749-016, Portugal; LASIGE, Faculdade de Ciências da Universidade de Lisboa, Campo Grande, Lisboa 1749-016, Portugal; International Centre for Cancer Vaccine Science, University of Gdansk, ul. Kładki 24, Gdańsk 80-822, Poland; International Centre for Cancer Vaccine Science, University of Gdansk, ul. Kładki 24, Gdańsk 80-822, Poland; LASIGE, Faculdade de Ciências da Universidade de Lisboa, Campo Grande, Lisboa 1749-016, Portugal; International Centre for Cancer Vaccine Science, University of Gdansk, ul. Kładki 24, Gdańsk 80-822, Poland; International Centre for Cancer Vaccine Science, University of Gdansk, ul. Kładki 24, Gdańsk 80-822, Poland; International Centre for Cancer Vaccine Science, University of Gdansk, ul. Kładki 24, Gdańsk 80-822, Poland; Department of Biochemistry and Microbiology, University of Victoria, 3800 Finnerty Rd, Victoria, British Columbia, BC V8P 5C2, Canada; Institute for Adaptive and Neural Computation, School of Informatics, University of Edinburgh, Old College, South Bridge, Edinburgh, EH8 9YL, UK; The Canadian Association for Responsible AI in Medicine, Victoria, Canada; LASIGE, Faculdade de Ciências da Universidade de Lisboa, Campo Grande, Lisboa 1749-016, Portugal

## Abstract

The adaptive immune response plays a vital role in eliminating infected and aberrant cells from the body. This process hinges on the presentation of short peptides by major histocompatibility complex Class I molecules on the cell surface. Immunopeptidomics, the study of peptides displayed on cells, delves into the wide variety of these peptides. Understanding the mechanisms behind antigen processing and presentation is crucial for effectively evaluating cancer immunotherapies. As an emerging domain, immunopeptidomics currently lacks standardization—there is neither an established terminology nor formally defined semantics—a critical concern considering the complexity, heterogeneity, and growing volume of data involved in immunopeptidomics studies. Additionally, there is a disconnection between how the proteomics community delivers the information about antigen presentation and its uptake by the clinical genomics community. Considering the significant relevance of immunopeptidomics in cancer, this shortcoming must be addressed to bridge the gap between research and clinical practice. In this work, we detail the development of the ImmunoPeptidomics Ontology, ImPO, the first effort at standardizing the terminology and semantics in the domain. ImPO aims to encapsulate and systematize data generated by immunopeptidomics experimental processes and bioinformatics analysis. ImPO establishes cross-references to 24 relevant ontologies, including the National Cancer Institute Thesaurus, Mondo Disease Ontology, Logical Observation Identifier Names and Codes and Experimental Factor Ontology. Although ImPO was developed using expert knowledge to characterize a large and representative data collection, it may be readily used to encode other datasets within the domain. Ultimately, ImPO facilitates data integration and analysis, enabling querying, inference and knowledge generation and importantly bridging the gap between the clinical proteomics and genomics communities. As the field of immunogenomics uses protein-level immunopeptidomics data, we expect ImPO to play a key role in supporting a rich and standardized description of the large-scale data that emerging high-throughput technologies are expected to bring in the near future.

**Ontology URL**: https://zenodo.org/record/10237571

**Project GitHub**: https://github.com/liseda-lab/ImPO/blob/main/ImPO.owl

## Introduction

The immunopeptidome refers to the various peptides presented on the cell surface by the immunoglobulin-like major histocompatibility complex (MHC) protein family (in humans, this family is also known by the name human leukocyte antigen or HLA) (1–3). T cells can detect mutated (nonself) MHC-associated peptides, triggering an immune response to eliminate the presenting cell. This immune response is driven by the paradigm of distinguishing between ‘self’ and ‘non-self’, which is significantly influenced by the antigen processing and presentation system.

Cancer immunotherapy and vaccine research have immensely benefited from an understanding of the immunopeptidomics landscape at various levels since the discovery of the human immunopeptidome allows the identification of vaccine and immunotherapy candidates, which could then be validated through clinical trials. It is known that cancer cells express aberrant immunopeptides on their surface that could be potentially recognized by T cells (3, 4); by studying these peptides and the mutations associated with them, it is possible to develop therapies that target those specific types of peptides on cancer cells, thus paving the way for personalized therapies that are highly specific to a particular tumor/patient.

Immunopeptidomics has proven to be a valuable tool in the literature, with several examples demonstrating its contribution to the discovery and development of new cancer therapies and vaccines. For instance, Singh-Jasuja *et al*. (5) developed the mass spectrometry (MS)-based XPRESIDENT platform, which led to the identification of multiple tumor-associated HLA-restricted epitopes and helped distinguish between HLA-restricted epitopes presented on healthy tumors and those on the surface of renal cell cancer tissue. As a direct result of their work, Walter *et al*. (6) developed IMA901, the first therapeutic vaccine against renal cell cancer. Carreno *et al*. (7) subjected three Stage III melanoma patients with known missense mutations in their tumors to a clinical trial for an experimental dendritic cell anti-melanoma vaccine, demonstrating the efficacy of immunopeptidomics approaches in detecting immune peptides of mutant origins within melanoma and other cancers. Nelde *et al*. (8) exploited immunopeptidomics data to develop a peptide vaccine ‘warehouse’, which could be used for the broad personalization of immunotherapy to patients diagnosed with various cancers, ultimately leading to the vaccine iVAC-XS15-Chronic Lymphocytic Leukemia (CLL) 01 against CLL. Hilf *et al*. (9) conducted the Glioma Actively Personalized Vaccine Consortium-101 trial to treat glioblastoma on 15 patients with the newly diagnosed disease through mutational and immunopeptidomics analysis. These studies illustrate the significance of data integration and analysis in successful immunopeptidomics research and its practical application.

Handling data produced by biomedical subdomains such as immunopeptidomics is a significant challenge due to its complexity, heterogeneity and volume. Comprehensively studying complex biological processes and improving knowledge discovery often require integrating several layers of (high-throughput) omics data with diverse phenotypic data (e.g. clinical data and medical images) and existing knowledge resources (10, 11). Among the latter are included ontologies such as the Gene Ontology (12) and databases such as Kyoto Encyclopedia of Genes and Genomes (13) or Reactome (14).

Effectively integrating biomedical data requires standardization in the form of an established common terminology for biomedical entities (e.g. gene names and anatomical parts) and formally defined semantics (i.e. the definitions of the entities and the relationships between them) (15–18). These are usually materialized as ontologies, representing knowledge in a domain by defining its entities, relations and potential attributes and asserting a common terminology (19, 20).

As an emerging domain, immunopeptidomics currently lacks standardization: there is neither an established terminology nor formally defined semantics. While there are already hundreds of ontologies spanning the biomedical domain—including neighbor domains such as proteomics (21) or immunology (22) or narrower subdomains such as immunogenomics (23) and immune epitopes (Ontology for Immune Epitopes and MHC) (24, 25)—none fully covers immunopeptidomics in either breadth or depth. The need for standardization is recognized by the community (26), and a set of guidelines representing the minimal information required to support the description of immunopeptidomics experiments Minimal Information About an Immuno-Peptidomics Experiment (MIAIPE) sufficiently has been developed (27). However, unlike some minimal information guidelines (28), MIAIPE does not prescribe which ontologies or controlled vocabularies should be employed to ensure that metadata values are objective, consistent and unambiguous across datasets. As a translational domain with critical relevance in cancer, this shortcoming must be addressed so that the research in this domain can quickly impact clinical practice and deliver on its promise (29, 30).

In this work, we detail the development of the ImmunoPeptidomics Ontology, ImPO (available at: https://github.com/liseda-lab/ImPO), the first effort at standardizing the terminology and semantics in the domain and supporting semantic data integration. ImPO was developed to serve as a component of a comprehensive knowledge graph (KG) that includes various biomedical ontologies to which ImPO was mapped. While these ontologies model concepts relevant to the immunopeptidomics domain, none adequately covers it in full, and most are not well suited to represent data directly, as they are class-centric and lacking in data properties. Thus, ImPO was developed to provide a data-centric view of immunopeptidomics and to be populated with actual data while also serving as a bridge between key domain ontologies that complement it and provide semantic depth.

ImPO was developed in the context of Knowledge At the Tip of Your fingers: Clinical Knowledge for Humanity (KATY), an European project aiming to bring ‘AI-empowered knowledge’ to the clinical practice using clear cell renal cell carcinoma as a pilot study (31). Together with the ontologies it is mapped to, ImPO forms the semantic layer of a personalized oncology KG (32), which will support data integration and provide explainability to the AI approaches developed in the project. Nevertheless, ImPO was also designed to be used independently from the KG as a standalone knowledge model to support data integration and knowledge discovery in immunopeptidomics.

## Background

‘Ontologies’ are formalizations of knowledge in a particular application domain, expressing concepts and their relationships in a manner that can be interpreted by both humans and computers. They serve as a source of standardized terminology and domain knowledge to deal with the challenges of data-intensive research, namely in the biomedical domain. We adopt the broad definition of what an ontology is proposed in (33), where ontologies may be viewed as a spectrum of detail in their specification, from simple controlled vocabularies to more complex artifacts which specify logical axioms and rules.

Ontologies are typically encoded in the Web Ontology Language (OWL), as recommended by the World Wide Web Consortium (W3C) (34). OWL is built on top of the Resource Description Framework (RDF) and therefore is characterized by its statements being triples of the form <subject> <predicate> <object> (even though this may not be readily apparent in some OWL serializations) and by the use of Internationalized Resource Identifiers to identify entities globally (34). OWL statements are axioms used to declare, characterize and relate the various entities in the application domain. OWL supports deductive reasoning, which is to say, the logical inference of non-stated facts based on the axioms asserted in the ontology (35).

Classes are conceptual representations of sets of individuals and can be related to each other through subclass relations or disjoint declarations. More complex non-hierarchical relations can be encoded through class expressions (e.g. ‘part of’ exactly 1 ‘gene’). Named individuals are unique and indivisible data-level entities representing concrete objects or instances within the scope of the ontology. Object properties represent relations between individuals and are used to connect them. Data properties represent attributes of individuals and are used to describe individuals with literals (or data values). Finally, annotation properties represent metadata attributes and are used to describe ontology entities for human readers, with properties such as ‘label’ and ‘hasExactSynonym’ accounting for the terminological component of ontologies (27). While ontologies can include a data layer (composed of individuals, their relations and attributes), when that layer is substantial, it is common to call them KGs (36, 37).

There are several approaches to ontology design, each with rules for selecting and expressing the terminology, its limitations and relations (38–41). According to Smirnov *et al*. (42), the ontology design process, analogously to database design, is typically a variation on the following general pattern: definition of requirements, conceptualization, implementation and evaluation.

At the definition of requirements stage, the intended application and scope of the ontology are identified and defined. During the conceptualization stage, domain knowledge is captured in collaboration with domain experts. Key concepts, relations and their constraints are identified and defined. Conceptual modeling techniques, such as entity–relationship (ER) modeling, are commonly employed at this stage to aid in structuring and representing the captured knowledge.

At the implementation stage, the domain knowledge captured previously is formalized in an ontology language such as OWL, often with the aid of an ontology editing tool, such as Protégé (42–44). At the evaluation stage, the ontology is assessed with respect to whether it fulfills all the requirements identified in the first stage and whether it is logically sound and conforms to good ontology design practices (45, 46).

In biomedical ontology design, it is common to integrate related ontologies. Formally, the mechanism for this integration should be through importing relevant ontologies, as per the W3C OWL guidelines (47), though this recommendation is often ignored in practice and isolated entities are directly reused from other ontologies without importing the ontologies (48). Alternatively, a lighter form of interoperability can be sought by declaring cross-references between related ontologies (i.e. annotations with property ‘hasDbXRef’).

Ontology matching plays a role in ontology design on the integration or interconnection of the new ontology with related ontologies, as it can be used to find matching entities in related ontologies (49). This is particularly critical in the biomedical domain, where there are hundreds of ontologies with overlapping domains (50–52).

It is crucial to evaluate a newly developed ontology before its publication or application, to ensure that it is complete with respect to the requirements for its intended application (45, 46) and that it is consistent both from a logical standpoint (explanation consistency) and with respect to good practices in ontology design (structural consistency) (16, 53–56).

One common strategy to assess the completeness of an ontology with respect to the application domain is using competency questions (CQs), i.e. questions formulated in natural language to be answered using data structured according to the ontology. Since these are questions with established or verifiable answers, they also function as content validation to determine if the ontology fits the application requirements and is structurally sound (57). Multiple CQs may be formulated to span different areas of the ontologies as well as specific parts of the data.

Explanation consistency is the absence of contradictory statements in the ontology (42, 43), which can be assessed using OWL reasoners. Structural consistency is concerned with aspects such as the naming of entities, the adequate assertion of class disjointness and property domains and ranges, and the definition of inverse object properties. It can be assessed with semi-automatic tools such as OOPS! (OntOlogy Pitfall Scanner!) (58).

## Materials and Methods

**Figure 1.**
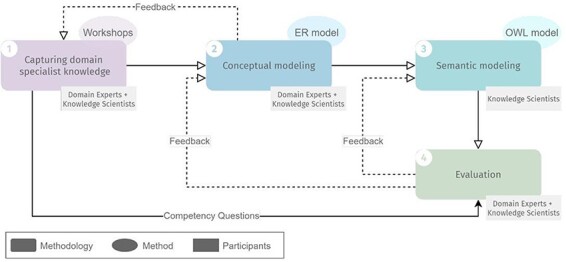
Overview of the methodology for developing ImPO, their methods and participants in each step. Full lines represent input from one step to another. Dashed lines represent feedback iteration cycles.

The design methodology we employed to design ImPO comprised four steps, schematized in Figure 1: (i) capturing domain specialist knowledge, (ii) conceptual modeling, (iii) semantic modeling and (iv) evaluation. Steps (i), (ii) and (iv) involved both domain experts and knowledge scientists, whereas step (iii) involved only the latter.

### Capturing domain specialist knowledge

The step of capturing domain specialist knowledge of our methodology corresponds to the traditional definition of the requirements stage of ontology design, but due to the specificity and complexity of the immunopeptidomics domain, it required several iterations interweaved with conceptual modeling. Domain experts shared immunopeptidomics domain knowledge with the knowledge scientists over a series of workshops, which encompassed lecture-type presentations, question-and-answer sessions and the analysis of immunopeptidomics experimental data files.

**Figure 2.**
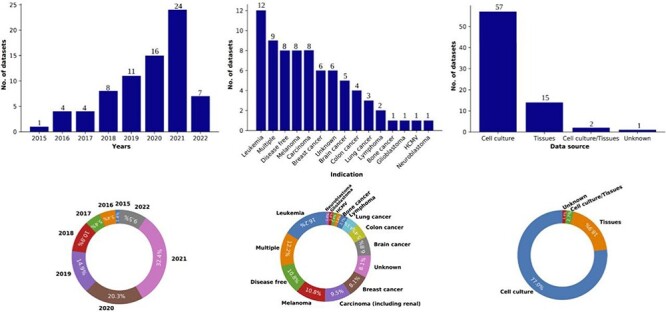
Overview of the data collected in the Knowledge At the Tip of Your (KATY) project. Distribution and percentage breakdown of datasets by year (left), disease condition (center) and data source (right). Total number of datasets collected (*n*) = 75.

Immunopeptidomics data are collected primarily through MS experiments (1, 59, 60) since this technique allows peptide antigens to be effectively eluted and detected in small amounts. Immunopeptidomics raw data are collected from the instrument as peaks/signals, subjected to denoising (to separate background noise from genuine signals), and wavelet transformation. The resulting data are defined by mainly two attributes, the peak height, or abundance, which is a measure of the intensity of an ion, and the mass-to-charge ratio of each ion, a measure of their charged states. Once peak and ion data have been obtained, the final step is annotating each peptide peak to its protein of origin, which is accomplished through database searches against standard protein databases such as ENSEMBL (61) or Uniprot (62) by validating the identification through various false discovery rate algorithms. Thus, immunopeptidomics data contain information about the peptide identified, its sequence, its length, its molecular weight, any post-translational modifications associated with it, the protein of origin and the spectra associated with the peptide among others. To realize the promise of immunopeptidomics and find effective targets for T-cell therapies or vaccine development, it is essential to connect the wealth of immunopeptidomics data to immunogenomics, establishing an integrated landscape. The immunopeptidomics data from the files used for the design of ImPO were collected between 2015 and 2022, numbering 75 datasets in total (Figure 2). Each dataset was downloaded from one of the two major proteomic data repositories: PRoteomics IDEntifications Database (PRIDE) (63) and Mass Spectrometry Interactive Virtual Environment (MassIVE) (64). A set of specific keywords were used to query the datasets (available in Supplementary File 1). The datasets corresponded to healthy and tumor conditions and were derived from cell culture, tissue and mixed sources (Table 1). The data were downloaded as raw files (containing raw spectra), and they were later processed through an in-house pipeline (65) to convert them into the Mascot generic file and mzML formats, which contained mass-to-charge and intensity/abundance data about each spectrum from the raw file. The total size of all datasets was 5.8 terabytes and was organized by PRIDE/MassIVE study id (the primary level) and sample/tissue (as the secondary/sub-level).

**Table 1.** Metadata from the downloaded datasets

S.No	Dataset_ID	Source	Source publication	Indication	MS instrument	Tissue	Year	Source
1	PXD014017	PRIDE	Newey A, Griffiths B, Michaux J, Pak HS, Stevenson BJ, Woolston A, Semiannikova M, Spain G, Barber LJ, Matthews N, Rao S, Watkins D, Chau I, Coukos G, Racle J, Gfeller D, Starling N, Cunningham D, Bassani-Sternberg M, Gerlinger M. Immunopeptidomics of colorectal cancer organoids reveals a sparse HLA class I neoantigen landscape and no increase in neoantigens with interferon or MEK-inhibitor treatment. J Immunother Cancer. 2019 7(1):309, PubMed: 31735170	Colon cancer	Q-Exactive	Colon	2019	Cell culture
2	PXD014397	PRIDE	Faridi P, Woods K, Ostrouska S, Deceneux C, Aranha R, Duscharla D, Wong SQ, Chen W, Ramarathinam SH, Lim Kam Sian TCC, Croft NP, Li C, Ayala R, Cebon JS, Purcell AW, Schittenhelm RB, Behren A. Spliced peptides and cytokine-driven changes in the immunopeptidome of melanoma. Cancer Immunol Res. 2020, PubMed: 32938616	Melanoma	Orbitrap	Skin	2020	Cell culture
3	PXD015039	PRIDE	Pandey K, Mifsud NA, Lim Kam Sian TCC, Ayala R, Ternette N, Ramarathinam SH, Purcell AW. In-depth mining of the immunopeptidome of an acute myeloid leukemia cell line using complementary ligand enrichment and data acquisition strategies. Mol Immunol. 2020 123:7–17, PubMed: 32387766	Leukemia	Orbitrap	Monocyte	2020	Cell culture
4	PXD015594	PRIDE	Kallenberger L, Erb R, Kralickova L, Patrignani A, Stöckli E, Jiricny J. Ectopic methylation of a single persistently unmethylated CpG in the promoter of the vitellogenin gene abolishes its inducibility by estrogen through attenuation of upstream stimulating factor binding. Mol Cell Biol. 2019 39(23), PubMed: 31548262	Carcinoma	Q-Exactive	Epithelial cell	2020	Cell culture
5	PXD015957	PRIDE	Kalaora S, Lee JS, Barnea E, Levy R, Greenberg P, Alon M, Yagel G, Bar Eli G, Oren R, Peri A, Patkar S, Bitton L, Rosenberg SA, Lotem M, Levin Y, Admon A, Ruppin E, Samuels Y. Immunoproteasome expression is associated with better prognosis and response to checkpoint therapies in melanoma. Nat Commun. 2020 11(1):896, PubMed: 32060274	Melanoma	Q-Exactive	Melanocyte	2020	Cell culture
6	PXD016060	PRIDE	Rijensky NM, Blondheim Shraga NR, Barnea E, Peled N, Rosenbaum E, Popovtzer A, Stemmer SM, Livoff A, Shlapobersky M, Moskovits N, Perry D, Rubin E, Haviv I, Admon A. Identification of tumor antigens in the HLA peptidome of patient-derived xenograft tumors in mouse. Mol Cell Proteomics. 2020, PubMed: 32451349	Multiple	Orbitrap/Q Exactive	Multiple	2020	Tissues
7	PXD016557	PRIDE	Not published	Carcinoma	Orbitrap/Q Exactive	Skin	2021	Cell culture
8	PXD017658	PRIDE	Not published	Carcinoma	Orbitrap/Q Exactive	Lung	2021	Cell culture
9	PXD017731	PRIDE	Do QT, Huang TE, Liu YC, Tai JH, Chen SH. Identification of cytosolic protein targets of catechol estrogens in breast cancer cells using a click chemistry-based workflow. J Proteome Res. 2020, PubMed: 32951420	Breast cancer	Orbitrap/Q Exactive	Breast	2020	Cell culture
10	PXD018124	PRIDE	Larouche JD, Trofimov A, Hesnard L, Ehx G, Zhao Q, Vincent K, Durette C, Gendron P, Laverdure JP, Bonneil É, Côté C, Lemieux S, Thibault P, Perreault C. Widespread and tissue-specific expression of endogenous retroelements in human somatic tissues. Genome Med. 2020 12(1):40, PubMed: 32345368	Carcinoma	Q-Exactive	Ovary	2020	Cell culture
11	PXD018539	PRIDE	Not published	Leukemia	Q-Exactive	Blood tissue	2021	Cell culture
12	PXD018540	PRIDE	Not published	Leukemia	Q-Exactive	Blood tissue	2021	Cell culture
13	PXD018541	PRIDE	Not published	Leukemia	Q-Exactive	Blood tissue	2021	Cell culture
14	PXD018542	PRIDE	Not published	Leukemia	Q-Exactive	Blood tissue	2021	Cell culture
15	PXD018543	PRIDE	Not published	Leukemia	Q-Exactive	Blood tissue	2021	Cell culture
16	PXD020079	PRIDE	Not published	Brain cancer	Q-Exactive	Brain	2021	Cell culture
17	PXD020111	PRIDE	Not published	Carcinoma	Orbitrap	Saliva	2020	Tissues
18	PXD020186	PRIDE	Marcu A, Bichmann L, Kuchenbecker L, Kowalewski DJ, Freudenmann LK, Backert L, Mühlenbruch L, Szolek A, Lübke M, Wagner P, Engler T, Matovina S, Wang J, Hauri-Hohl M, Martin R, Kapolou K, Walz JS, Velz J, Moch H, Regli L, Silginer M, Weller M, Löffler MW, Erhard F, Schlosser A, Kohlbacher O, Stevanoviæ S, Rammensee HG, Neidert MC. HLA Ligand Atlas: a benign reference of HLA-presented peptides to improve T-cell-based cancer immunotherapy. J Immunother Cancer. 2021 9(4), PubMed: 33858848	Brain cancer	Orbitrap	Brain	2021	Tissues
19	PXD020211	PRIDE	Not published	Carcinoma	Orbitrap	Saliva	2020	Tissues
20	PXD020224	PRIDE	Bartok O, Pataskar A, Nagel R, Laos M, Goldfarb E, Hayoun D, Levy R, Körner PR, Kreuger IZM, Champagne J, Zaal EA, Bleijerveld OB, Huang X, Kenski J, Wargo J, Brandis A, Levin Y, Mizrahi O, Alon M, Lebon S, Yang W, Nielsen MM, Stern-Ginossar N, Altelaar M, Berkers CR, Geiger T, Peeper DS, Olweus J, Samuels Y, Agami R. Anti-tumour immunity induces aberrant peptide presentation in melanoma. Nature. 2021 590(7845):332–337, PubMed: 33328638	Melanoma	Orbitrap/Q-Exactive	Skin	2020	Cell culture
21	PXD021270	PRIDE	Bressan RB, Southgate B, Ferguson KM, Blin C, Grant V, Alfazema N, Wills JC, Marques-Torrejon MA, Morrison GM, Ashmore J, Robertson F, Williams CAC, Bradley L, von Kriegsheim A, Anderson RA, Tomlinson SR, Pollard SM. Regional identity of human neural stem cells determines oncogenic responses to histone H3.3 mutants. Cell Stem Cell. 2021 28(5):877–893.e9, PubMed: 33631116	Brain cancer	Q-Exactive	Brain	2021	Cell culture
22	PXD021755	PRIDE	Not published	Colon cancer	Orbitrap	Colon	2021	Cell culture
23	PXD023038	PRIDE	Not published	Breast cancer	Orbitrap	Breast	2021	Cell culture
24	PXD023044	PRIDE	Not published	Breast cancer	Orbitrap	Breast	2021	Cell culture
25	PXD023064	PRIDE	Not published	Colon cancer	Orbitrap	Colon	2021	Cell culture
26	PXD024917	PRIDE	Stopfer LE, Gajadhar AS, Patel B, Gallien S, Frederick DT, Boland GM, Sullivan RJ, White FM. Absolute quantification of tumor antigens using embedded MHC-I isotopologue calibrants. Proc Natl Acad Sci U S A. 2021 118(37), PubMed: 34497125	Unknown	Orbitrap/Q-Exactive	Skin	2021	Cell culture
27	PXD027182	PRIDE	Yarmarkovich M, Marshall QF, Warrington JM, Premaratne R, Farrel A, Groff D, Li W, di Marco M, Runbeck E, Truong H, Toor JS, Tripathi S, Nguyen S, Shen H, Noel T, Church NL, Weiner A, Kendsersky N, Martinez D, Weisberg R, Christie M, Eisenlohr L, Bosse KR, Dimitrov DS, Stevanovic S, Sgourakis NG, Kiefel BR, Maris JM. Cross-HLA targeting of intracellular oncoproteins with peptide-centric CARs. Nature. 2021 599(7885):477–484, PubMed: 34732890	Neuroblastoma	Orbitrap	Kidney	2021	Cell culture
28	PXD004746	PRIDE	Khodadoust MS, Olsson N, Wagar LE, Haabeth OA, Chen B, Swaminathan K, Rawson K, Liu CL, Steiner D, Lund P, Rao S, Zhang L, Marceau C, Stehr H, Newman AM, Czerwinski DK, Carlton VE, Moorhead M, Faham M, Kohrt HE, Carette J, Green MR, Davis MM, Levy R, Elias JE, Alizadeh AA. Antigen presentation profiling reveals recognition of lymphoma immunoglobulin neoantigens. Nature. 22 March 2017, PubMed: 28329770	Lymphoma	Orbitrap	Blood/mononuclear cells/bone marrow	2017	Cell culture
29	PXD012308	PRIDE	Racle J, Michaux J, Rockinger GA, Arnaud M, Bobisse S, Chong C, Guillaume P, Coukos G, Harari A, Jandus C, Bassani-Sternberg M, Gfeller D. Robust prediction of HLA class II epitopes by deep motif deconvolution of immunopeptidomes. Nat Biotechnol. 2019, PubMed: 31611696	Multiple	Q-Exactive	Unknown	2019	Cell culture
30	PXD011628	PRIDE	Bichmann L, Nelde A, Ghosh M, Heumos L, Mohr C, Peltzer A, Kuchenbecker L, Sachsenberg T, Walz JS, Stevanoviæ S, Rammensee HG, Kohlbacher O. MHCquant: Automated and reproducible data analysis for immunopeptidomics. J Proteome Res. 2019, PubMed: 31589052; Bassani-Sternberg M, Bräunlein E, Klar R, Engleitner T, Sinitcyn P, Audehm S, Straub M, Weber J, Slotta-Huspenina J, Specht K, Martignoni ME, Werner A, Hein R, H Busch D, Peschel C, Rad R, Cox J, Mann M, Krackhardt AM. Direct identification of clinically relevant neoepitopes presented on native human melanoma tissue by mass spectrometry. Nat Commun. 2016 7:13404, PubMed: 27869121	Disease free	Q-Exactive	Blood tissue	2019	Cell culture
31	PXD012083	PRIDE	Narayan R, Olsson N, Wagar LE, Medeiros BC, Meyer E, Czerwinski D, Khodadoust MS, Zhang L, Schultz L, Davis MM, Elias JE, Levy R. Acute myeloid leukemia immunopeptidome reveals HLA presentation of mutated nucleophosmin. PLoS One. 2019 14(7):e0219547, PubMed: 31291378	Leukemia	Orbitrap	Blood tissue	2019	Cell culture/tissues
32	PXD011766	PRIDE	Koumantou D, Barnea E, Martin-Esteban A, Maben Z, Papakyriakou A, Mpakali A, Kokkala P, Pratsinis H, Georgiadis D, Stern LJ, Admon A, Stratikos E. Editing the immunopeptidome of melanoma cells using a potent inhibitor of endoplasmic reticulum aminopeptidase 1 (ERAP1). Cancer Immunol Immunother. 2019, PubMed: 31222486	Melanoma	Q-Exactive	Skin	2019	Cell culture
33	PXD013057	PRIDE	Löffler MW, Mohr C, Bichmann L, Freudenmann LK, Walzer M, Schroeder CM, Trautwein N, Hilke FJ, Zinser RS, Mühlenbruch L, Kowalewski DJ, Schuster H, Sturm M, Matthes J, Riess O, Czemmel S, Nahnsen S, Königsrainer I, Thiel K, Nadalin S, Beckert S, Bösmüller H, Fend F, Velic A, Maèek B, Haen SP, Buonaguro L, Kohlbacher O, Stevanoviæ S, Königsrainer A, HEPAVAC Consortium, Rammensee HG. Multi-omics discovery of exome-derived neoantigens in hepatocellular carcinoma. Genome Med. 2019 11(1):28, PubMed: 31039795	Carcinoma	Orbitrap	Liver	2019	Tissues
34	PXD011723	PRIDE	Andreatta M, Nicastri A, Peng X, Hancock G, Dorrell L, Ternette N, Nielsen M. MS-rescue: A Computational pipeline to increase the quality and yield of immunopeptidomics experiments. Proteomics. 2018:e1800357, PubMed: 30578603	Unknown	Orbitrap	Blood tissue	2019	Cell culture
35	PXD007203	PRIDE	Erhard F, Halenius A, Zimmermann C, L’Hernault A, Kowalewski DJ, Weekes MP, Stevanovic S, Zimmer R, Dölken L. Improved Ribo-seq enables identification of cryptic translation events. Nat Methods. 2018, PubMed: 29529017	Human cytomegalovirus	Orbitrap	Foreskin	2018	Cell culture
36	PXD004233	PRIDE	Mommen GP, Frese CK, Meiring HD, van Gaans-van den Brink J, de Jong AP, van Els CA, Heck AJ; Expanding the detectable HLA peptide repertoire using electron-transfer/higher-energy collision dissociation (EThcD). Proc Natl Acad Sci U S A, 25 March 2014, 111, 12, 4507–12, PubMed: 24616531; Marino F, Mommen GP, Jeko A, Meiring HD, van Gaans-van den Brink JA, Scheltema RA, van Els CA, Heck AJ. Arginine (di)methylated Human Leukocyte Antigen class I peptides are favorably presented by HLA-B*07. J Proteome Res. 8 August 2016, PubMed: 27503676	Disease free	Orbitrap	Unknown	2016	Cell culture
37	PXD003790	PRIDE	Shraibman B, Melamed Kadosh D, Barnea E, Admon A. HLA peptides derived from tumor antigens induced by inhibition of DNA methylation for development of drug-facilitated immunotherapy. Mol Cell Proteomics. 13 July 2016. pii: mcp.M116.060350, PubMed: 27412690	Glioblastoma	Q-Exactive	Brain	2016	Cell culture
38	MSV000080527	MassIVE	Abelin JG, Keskin DB, Sarkizova S, Hartigan CR, Zhang W, Sidney J, Stevens J, Lane W, Zhang GL, Eisenhaure T, Clauser KR, Hacohen N, Rooney MS, Carr SA, and Wu, CJ. Immunity, 2017	Multiple	Q-Exactive	Multiple	2020	Cell culture
39	MSV000084172	MassIVE	Abelin JG, Keskin DB, Sarkizova S, Hartigan CR, Zhang W, Sidney J, Stevens J, Lane W, Zhang GL, Eisenhaure T, Clauser KR, Hacohen N, Rooney MS, Carr SA, and Wu, CJ. Immunity, 2018	Multiple	Q-Exactive	Multiple	2020	Cell culture
40	MSV000084442	MassIVE	Abelin JG, Keskin DB, Sarkizova S, Hartigan CR, Zhang W, Sidney J, Stevens J, Lane W, Zhang GL, Eisenhaure T, Clauser KR, Hacohen N, Rooney MS, Carr SA, and Wu, CJ. Immunity, 2019	Multiple	Q-Exactive	Multiple	2020	Cell culture
41	PXD001898-PASS00270	PRIDE	Laumont CM, Daouda T, Laverdure JP, Bonneil É, Caron-Lizotte O, Hardy MP, Granados DP, Durette C, Lemieux S, Thibault P, Perreault C. Global proteogenomic analysis of human MHC class I-associated peptides derived from non-canonical reading frames. Nat Commun. 5 January 2016;7:10238, PubMed: 26728094	Disease free	Orbitrap	B lymphocyte	2016	Cell culture
42	PXD007860	PRIDE	Olsson N, Schultz LM, Zhang L, Khodadoust MS, Narayan R, Czerwinski DK, Levy R, Elias JE. T-cell immunopeptidomes reveal cell subtype surface markers derived from intracellular proteins. Proteomics. 2018, PubMed: 29493099	Disease free	Orbitrap	Blood/T lymphocyte	2018	Cell culture
43	PXD011257	PRIDE	Demmers LC, Heck AJR, Wu W. Pre-fractionation extends, but also creates a bias in the detectable HLA class I ligandome. J Proteome Res. 2019, PubMed: 30784271	Disease free	Orbitrap	B lymphocyte	2019	Cell culture
44	PXD007935	PRIDE	Lanoix J, Durette C, Courcelles M, Cossette É, Comtois-Marotte S, Hardy MP, Côté C, Perreault C, Thibault P. Comparison of the MHC I immunopeptidome repertoire of B-cell lymphoblasts using two isolation methods. Proteomics. 2018, PubMed: 29508533	Leukemia	Q-Exactive	Spleen	2018	Cell culture
45	PXD009752	PRIDE	Laumont CM, Vincent K, Hesnard L, Audemard É, Bonneil É, Laverdure JP, Gendron P, Courcelles M, Hardy MP, Côté C, Durette C, St-Pierre C, Benhammadi M, Lanoix J, Vobecky S, Haddad E, Lemieux S, Thibault P, Perreault C. Noncoding regions are the main source of targetable tumor-specific antigens. Sci Transl Med. 2018 10(470), PubMed: 30518613	Lung cancer	Q-Exactive	Lung	2018	Tissues
46	PXD009754	PRIDE	Laumont CM, Vincent K, Hesnard L, Audemard É, Bonneil É, Laverdure JP, Gendron P, Courcelles M, Hardy MP, Côté C, Durette C, St-Pierre C, Benhammadi M, Lanoix J, Vobecky S, Haddad E, Lemieux S, Thibault P, Perreault C. Noncoding regions are the main source of targetable tumor-specific antigens. Sci Transl Med. 2018 10(470), PubMed: 30518613	Lung cancer	Q-Exactive	Lung	2019	Tissues
47	PXD009755	PRIDE	Laumont CM, Vincent K, Hesnard L, Audemard É, Bonneil É, Laverdure JP, Gendron P, Courcelles M, Hardy MP, Côté C, Durette C, St-Pierre C, Benhammadi M, Lanoix J, Vobecky S, Haddad E, Lemieux S, Thibault P, Perreault C. Noncoding regions are the main source of targetable tumor-specific antigens. Sci Transl Med. 2018 10(470), PubMed: 30518613	Lung cancer	Q-Exactive	Lung	2020	Tissues
48	PXD004023	PRIDE	Pearson H, Daouda T, Granados DP, Durette C, Bonneil E, Courcelles M, Rodenbrock A, Laverdure JP, Côté C, Mader S, Lemieux S, Thibault P, Perreault C. MHC class I-associated peptides derive from selective regions of the human genome. J Clin Invest. 1 December 2016;126(12):4690–4701, PubMed: 27841757	Disease free	Orbitrap/Q-Exactive	B lymphocyte	2016	Cell culture
49	PXD007596	PRIDE	Komov L, Kadosh DM, Barnea E, Milner E, Hendler A, Admon A. Cell surface MHC class I expression is limited by the availability of peptide-receptive ‘empty’ molecules rather than by the supply of peptide ligands. Proteomics. 2018:e1700248, PubMed: 29707912	Breast cancer	Q-Exactive	Epithelial cell	2018	Cell culture
50	PXD009531	PRIDE	Di Marco M, Schuster H, Backert L, Ghosh M, Rammensee HG, Stevanoviæ S. Unveiling the peptide motifs of HLA-C and HLA-G from naturally presented peptides and generation of binding prediction matrices. J Immunol. 2017 199(8):2639–2651, PubMed: 28904123	Disease free	Orbitrap	Leukocyte	2018	Cell culture
51	PXD010808	PRIDE	Khodadoust MS, Olsson N, Chen B, Sworder B, Shree T, Liu CL, Zhang L, Czerwinski DK, Davis MM, Levy R, Elias JE, Alizadeh AA. B cell lymphomas present immunoglobulin neoantigens. Blood. 2018, PubMed: 30545830	Lymphoma	Orbitrap	B lymphocyte	2019	Cell culture
52	PXD008937	PRIDE	Zeiner PS, Zinke J, Kowalewski DJ, Bernatz S, Tichy J, Ronellenfitsch MW, Thorsen F, Berger A, Forster MT, Muller A, Steinbach JP, Beschorner R, Wischhusen J, Kvasnicka HM, Plate KH, Stefanoviæ S, Weide B, Mittelbronn M, Harter PN. CD74 regulates complexity of tumor cell HLA class II peptidome in brain metastasis and is a positive prognostic marker for patient survival. Acta Neuropathol Commun. 2018 6(1):18, PubMed: 29490700	Brain cancer	Orbitrap	Skin	2018	Cell culture
53	PXD009738	PRIDE	Ternette N, Olde Nordkamp MJM, Müller J, Anderson AP, Nicastri A, Hill AVS, Kessler BM, Li D. Immunopeptidomic profiling of HLA-A2-positive triple negative breast cancer identifies potential immunotherapy target antigens. Proteomics. 2018 18(12):e1700465, PubMed: 29786170	Breast cancer	Orbitrap	Breast	2018	Tissues
54	PXD006939	PRIDE	Chong C, Marino F, Pak H, Racle J, Daniel RT, Müller M, Gfeller D, Coukos G, Bassani-Sternberg M. High-throughput and sensitive immunopeptidomics platform reveals profound interferonγ -mediated remodeling of the human leukocyte antigen (HLA) ligandome. Mol Cell Proteomics. 2018 17(3):533–548, PubMed: 29242379	Multiple	Q-Exactive	B/T lymphocyte	2017	Cell culture
55	PXD005231	PRIDE	Not published	Melanoma	Orbitrap/Q-Exactive	B/T lymphocyte	2017	Cell culture
56	PXD000394	PRIDE	Bassani-Sternberg M, Pletscher-Frankild S, Jensen LJ, Mann M. Mass spectrometry of human leukocyte antigen class I peptidomes reveals strong effects of protein abundance and turnover on antigen presentation. Mol Cell Proteomics. 2015 Mar;14(3):658–73, PubMed: 25576301	Multiple	Q-Exactive	B lymphocyte	2015	Cell culture
57	PXD004894	PRIDE	Bassani-Sternberg M, Bräunlein E, Klar R, Engleitner T, Sinitcyn P, Audehm S, Straub M, Weber J, Slotta-Huspenina J, Specht K, Martignoni ME, Werner A, Hein R, H Busch D, Peschel C, Rad R, Cox J, Mann M, Krackhardt AM. Direct identification of clinically relevant neoepitopes presented on native human melanoma tissue by mass spectrometry. Nat Commun. 21 November 2016;7:13404, PubMed: 27869121	Melanoma	Q-Exactive	Melanocyte	2017	Tissues
58	PXD028921	PRIDE	Pataskar A, Champagne J, Nagel R, Kenski J, Laos M, Michaux J, Pak HS, Bleijerveld OB, Mordente K, Navarro JM, Blommaert N, Nielsen MM, Lovecchio D, Stone E, Georgiou G, de Gooijer MC, van Tellingen O, Altelaar M, Joosten RP, Perrakis A, Olweus J, Bassani-Sternberg M, Peeper DS, Agami R. Tryptophan depletion results in tryptophan-to-phenylalanine substitutants. Nature. 2022 603(7902):721–727, PubMed: 35264796	Melanoma	Orbitrap/Q-Exactive	Melanocyte	2021	Cell culture
59	PXD028874	PRIDE	Not published	Unknown	Orbitrap	B lymphocyte	2021	Cell culture
60	PXD025655	PRIDE	Parker R, Tailor A, Peng X, Nicastri A, Zerweck J, Reimer U, Wenschuh H, Schnatbaum K, Ternette N. The choice of search engine affects sequencing depth and HLA class I allele-specific peptide repertoires. Mol Cell Proteomics. 2021:100124, PubMed: 34303857	Multiple	Q-Exactive	Epithelial cell	2021	Cell culture
61	PXD025073	PRIDE	Stopfer LE, Conage-Pough JE, White FM. Quantitative consequences of protein carriers in immunopeptidomics and tyrosine phosphorylation MS^2^ analyses. Mol Cell Proteomics. 2021:100104, PubMed: 34052394	Unknown	Orbitrap	Multiple	2021	Cell culture
62	PXD024562	PRIDE	Not published	Unknown	Orbitrap	Unknown	2021	Cell culture
63	PXD019676	PRIDE	Not published	Breast cancer	Q-Exactive	Breast	2022	Cell culture
64	PXD033340	PRIDE	Garg SK, Welsh EA, Fang B, Hernandez YI, Rose T, Gray J, Koomen JM, Berglund A, Mulé JJ, Markowitz J. Multi-omics and informatics analysis of FFPE tissues derived from melanoma patients with long/short responses to anti-PD1 therapy reveals pathways of response. Cancers (Basel). 2020 12(12), PubMed: 33255891	Melanoma	Q-Exactive	Unknown	2022	Tissues
65	PXD030166	PRIDE	Huiting W, Dekker SL, van der Lienden JCJ, Mergener R, Musskopf MK, Furtado GV, Gerrits E, Coit D, Oghbaie M, Di Stefano LH, Schepers H, van Waarde-Verhagen MAWH, Couzijn S, Barazzuol L, LaCava J, Kampinga HH, Bergink S. Targeting DNA topoisomerases or checkpoint kinases results in an overload of chaperone systems, triggering aggregation of a metastable subproteome. Elife. 2022 11:e70726, PubMed: 35200138	Bone cancer	Orbitrap	Unknown	2022	Cell culture
66	PXD031709	PRIDE	not published	Unknown	Orbitrap	Unknown	2022	Unknown
67	PXD019643	PRIDE	Marcu A, Bichmann L, Kuchenbecker L, Kowalewski DJ, Freudenmann LK, Backert L, Mühlenbruch L, Szolek A, Lübke M, Wagner P, Engler T, Matovina S, Wang J, Hauri-Hohl M, Martin R, Kapolou K, Walz JS, Velz J, Moch H, Regli L, Silginer M, Weller M, Löffler MW, Erhard F, Schlosser A, Kohlbacher O, Stevanoviæ S, Rammensee HG, Neidert MC. HLA Ligand Atlas: a benign reference of HLA-presented peptides to improve T-cell-based cancer immunotherapy. J Immunother Cancer. 2021 9(4), PubMed: 33858848	Disease free	Orbitrap	Multiple	2021	Tissues
68	PXD020186	PRIDE	Marcu A, Bichmann L, Kuchenbecker L, Kowalewski DJ, Freudenmann LK, Backert L, Mühlenbruch L, Szolek A, Lübke M, Wagner P, Engler T, Matovina S, Wang J, Hauri-Hohl M, Martin R, Kapolou K, Walz JS, Velz J, Moch H, Regli L, Silginer M, Weller M, Löffler MW, Erhard F, Schlosser A, Kohlbacher O, Stevanoviæ S, Rammensee HG, Neidert MC. HLA Ligand Atlas: a benign reference of HLA-presented peptides to improve T-cell-based cancer immunotherapy. J Immunother Cancer. 2021 9(4), PubMed: 33858848	Brain cancer	Orbitrap	Brain	2021	Tissues
69	PXD028309	PRIDE	Cleyle J, Hardy MP, Minati R, Courcelles M, Durette C, Lanoix J, Laverdure JP, Vincent K, Perreault C, Thibault P. Immunopeptidomic analyses of colorectal cancers with and without microsatellite instability. Mol Cell Proteomics. 2022:100228, PubMed: 35367648	Colon cancer	Orbitrap	Colon	2022	Cell culture/tissues
70	PXD025716	PRIDE	Nelde A, Flötotto L, Jürgens L, Szymik L, Hubert E, Bauer J, Schliemann C, Kessler T, Lenz G, Rammensee HG, Walz JS, Wethmar K. Upstream open reading frames regulate translation of cancer-associated transcripts and encode HLA-presented immunogenic tumor antigens. Cell Mol Life Sci. 2022 79(3):171, PubMed: 35239002	Multiple	Orbitrap	Multiple	2022	Tissues
71	PXD009749	PRIDE	Laumont CM, Vincent K, Hesnard L, Audemard É, Bonneil É, Laverdure JP, Gendron P, Courcelles M, Hardy MP, Côté C, Durette C, St-Pierre C, Benhammadi M, Lanoix J, Vobecky S, Haddad E, Lemieux S, Thibault P, Perreault C. Noncoding regions are the main source of targetable tumor-specific antigens. Sci Transl Med. 2018 10(470), PubMed: 30518613	Leukemia	Q-Exactive	Spleen	2019	Cell culture
72	PXD009753	PRIDE	Laumont CM, Vincent K, Hesnard L, Audemard É, Bonneil É, Laverdure JP, Gendron P, Courcelles M, Hardy MP, Côté C, Durette C, St-Pierre C, Benhammadi M, Lanoix J, Vobecky S, Haddad E, Lemieux S, Thibault P, Perreault C. Noncoding regions are the main source of targetable tumor-specific antigens. Sci Transl Med. 2018 10(470), PubMed: 30518613	Leukemia	Q-Exactive	Spleen	2020	Cell culture
73	PXD009750	PRIDE	Laumont CM, Vincent K, Hesnard L, Audemard É, Bonneil É, Laverdure JP, Gendron P, Courcelles M, Hardy MP, Côté C, Durette C, St-Pierre C, Benhammadi M, Lanoix J, Vobecky S, Haddad E, Lemieux S, Thibault P, Perreault C. Noncoding regions are the main source of targetable tumor-specific antigens. Sci Transl Med. 2018 10(470), PubMed: 30518613	Leukemia	Q-Exactive	Spleen	2021	Cell culture
74	PXD009751	PRIDE	Laumont CM, Vincent K, Hesnard L, Audemard É, Bonneil É, Laverdure JP, Gendron P, Courcelles M, Hardy MP, Côté C, Durette C, St-Pierre C, Benhammadi M, Lanoix J, Vobecky S, Haddad E, Lemieux S, Thibault P, Perreault C. Noncoding regions are the main source of targetable tumor-specific antigens. Sci Transl Med. 2018 10(470), PubMed: 30518614	Leukemia	Q-Exactive	Spleen	2022	Cell culture
75	PXD017149	PRIDE	Reustle A, Di Marco M, Meyerhoff C, Nelde A, Walz JS, Winter S, Kandabarau S, Büttner F, Haag M, Backert L, Kowalewski DJ, Rausch S, Hennenlotter J, Stühler V, Scharpf M, Fend F, Stenzl A, Rammensee HG, Bedke J, Stevanoviæ S, Schwab M, Schaeffeler E. Integrative -omics and HLA-ligandomics analysis to identify novel drug targets for ccRCC immunotherapy. Genome Med. 2020 12(1):32, PubMed: 32228647	Carcinoma	Orbitrap	Kidney	2020	Tissues

The processed immunopeptidomics data was distributed into several files, 10 of which were relevant to the design of ImPO and are listed in Table 2. More information about the source data is available in (65). (The listed datasets listed have also been uploaded onto a Figshare repository accessible through the URL Figshare repository named ‘ImPo Table 2 Datasets’: https://figshare.com/s/69e32bea6c69055d7693.) Additional data were collected from the Catalogue of Somatic Mutations in Cancer database (a publicly available dataset of cancer mutations called across cancers, version 96, https://cancer.sanger.ac.uk/cosmic).

**Table 2.** Brief description of the immunopeptidomics data files and their most relevant data

Data file name	Description	Relevant data
0.05_LG_umap_peptides_annotation_clustering	Information about peptides annotated with position-specific weight matrix (PSWM) clustering	Peptide sequence, sample ID
pswm_clustering_annotation	Information about PSWM clustering annotation	Components 1 and 2, pswm group, inferred allele, and sample ID
closed_search_PSMs_HLA-I	Information obtained through closed search MS strategy	Spectrum, peptide sequence, sample ID, PRIDE ID (study ID), score
denovo_90ALC_HLA-I	Information obtained through de novo MS strategy	Spectrum, peptide sequence, sample ID, PRIDE ID, score
opensearch_PTMiner_HLA-I	Information obtained through open search MS strategy	Spectrum, peptide sequence, sample ID, PRIDE ID, score, position, mass shift, annotated modification and type
CosmicMutantExport_nonredundant-immune-visible	Information about genomic mutations from COSMIC (79)	Mutation strand, chromosome, start and end, contig, mutation ID
GenomicImmuneClusters	Information about the Epitope Contigs (previously named Genomic Immune Clusters or Genomic hotspots)	Epitope contig id, chromosome, strand, start, end, gene mutational ratio, population coverage, overlap score, expression, and immune score
immunopeptides_library	Information about all the peptides identified	Peptide sequence, start, end, chromosome, tag, MS analysis strategy, sample ID, and study ID
mappings	Information about the genes and their products (transcripts and proteins)	Gene name and ID, transcript name and ID, protein ID
Supplementary_table_1	Information and details about the studies from where the information was extracted	Cancer type, Sample ID, type, and treatment, MS instrument, study ID

Once the knowledge scientists had acquired sufficient knowledge of the main domain concepts and were able to interpret the data files, a first draft of an ER conceptual model for immunopeptidomics was produced, and subsequent workshops focused on revising and refining this model, often revisiting the data files. In total, 11 workshops, with a duration of 1–2 hours, were held to capture domain knowledge and/or refine the ER conceptual model. Further capturing of knowledge from the domain experts took place via e-mail exchanges, in particular for the definition of CQs for the evaluation stage.

### Conceptual modeling

The ER model was chosen as the data model for our conceptual modeling step as it is sufficiently intuitive to enable the domain specialists to understand and revise the resulting model while sufficiently structured and expressive to capture the semantics of the domain and serve as the foundation for an OWL ontology. Our approach to conceptual modeling was initially top-down and, as stated in the preceding section, iterative and interwoven with the step of capturing domain specialist knowledge. We used the web application diagramsnet () to design our ER model.

We started by conceptually dividing the immunopeptidomics domain into two main subdomains: the biological, containing entities such as patients, diseases, genes and mutations; and the experimental, containing entities such as the studies and assays whereby biological entities were assessed. The latter was critical not only to fully model the immunopeptidomics data but, more importantly, to enable traceability of the biological assertions. We modeled in ER the core concepts and their main attributes in each subdomain and the relationships between them, as well as the relationships between concepts of the two subdomains. We then extended the preliminary ER model through analysis of the data files, adding the entities, relationships and attributes necessary to model them and where necessary revising previously defined entities or relationships. This first draft of the ER model for the immunopeptidomics domain was reviewed by the domain experts, which often elicited further capturing of domain specialist knowledge through the workshops.

When no more alterations to the ER model were required by the domain experts, we deemed it sufficiently mature to start the process of transformation into OWL. However, the evaluation of the OWL ontology later revealed aspects of the ER model which were not entirely accurate, eliciting further revisions.

### Semantic modeling

We chose OWL as the language to express ImPO as it is the standard recommended by the W3C and used the ontology editor Protégé (43) to manually execute the conversion of the ER model into OWL. With respect to expressiveness, we elected to conform to the syntactic restrictions required to make ImPO an OWL 2 DL ontology, which is the most expressive subset of OWL that is supported by current OWL reasoners. This decision was made upon realization that none of the ‘lighter’ subsets of OWL was sufficiently expressive to accurately model the immunopeptidomics domain and data, and although even conforming to OWL 2 DL required minor sacrifices in expressiveness, it was critical to enable reasoning, which is key to support ontology-based querying of the data—the main reason behind the development of ImPO.

Our conversion of the ER model to OWL followed the general guidelines listed in the Converting ER to OWL subsection. We started by converting ER entities to OWL classes, then defined the OWL data properties and data property restrictions on the classes that corresponded to the ER attributes of the entities, and finally analyzed the ER relationships and converted them into either OWL object properties or aggregates of an OWL class plus 2+ OWL object properties plus 0+ OWL data properties as necessary to model the arity and attributes of the ER relation. Object property restrictions on the classes were defined as necessary to model cardinality and participation restrictions on the ER relations.

Regarding the naming convention in ImPO, we opted to use human-readable local names corresponding to the labels but with underscores (e.g. ‘human_leukocyte_antigen’) as recommended by the OWL guidelines rather than the alpha-numeric codes more common in the biomedical domain (e.g. ‘NCIT_C80488’). This decision was motivated by the fact that ImPO is geared toward directly describing data by instancing the ontology, in the interest of making such data human readable. Had we used alpha-numeric codes, interpreting the data would require constant consultation of the label corresponding to each code. In addition to labels, all classes in ImPO were annotated with a textual definition provided by the domain experts using the ‘rdfs:comment’ property.

With respect to integrating ImPO with existing biomedical ontologies, we contemplated two potential approaches: formal integration by extending a related broader ontology, such as the National Cancer Institute Thesaurus (NCIt) (67) or a sister ontology, such as Immunogenetics Ontology (23), with the concepts that are specific to immunopeptidomics; and informal integration through cross-references to related ontologies. We opted for the latter due to the fundamental difference in scope between ImPO (which aims to be instanced with data) and existing biomedical ontologies (which are geared toward data annotation and classification). Due to these differences, had we opted to extend an existing ontology, data representation would be unnecessarily complex and/or not fully accurate, and we would have to modify definitions of entities imported from other ontologies. The cross-referencing approach allowed us to model the data directly and accurately in a self-contained manner while supporting interoperability at the conceptual level through the cross-references. Moreover, the cross-references also serve as anchors for integrating ImPO with key domain ontologies under the KATY KG.

The cross-referencing process was mediated by the ontology matching tool AgreementMakerLight (68), which was used automatically, with default settings, to map ImPO to the 28 key domain ontologies identified by domain experts in the KATY project (32) and two additional ontologies from adjacent domains, Immunogenetics Ontology (23) and Ontology for Immune Epitopes (24). The resulting alignments were manually revised, with incorrect and duplicated mappings removed. The mappings deemed correct were incorporated into ImPO in the form of cross-references, programmatically with the OWL API (69).

### Evaluation

Once the semantic modeling process was finalized, we evaluated the ontology with respect to both consistency and completeness.

Explanation (or logical) consistency was assessed through the use of the automated reasoner HermiT (version 1.4.3.456) in Protégé, which also served to ensure ImPO conformed to the OWL 2 DL subset of OWL, as this is a prerequisite for the reasoner. Structural consistency was assessed through the use of a web application (https://oops.linkeddata.es/, accessed 16 October 2022) based on OOPS! (58), which analyzes 41 common pitfalls and ranks them according to their impact on ontology quality: critical, important and minor. OOPS! was chosen over other tools, such as ROBOT (70) due to the fact that ROBOT is focused on optimizing the development of large-class hierarchies for data annotation purposes.

Completeness was assessed by the means of 15 CQs formulated by the domain experts. Assessing it required: (i) populating the ontology with data to which the CQs could be applied; (ii) encoding the CQs in SPARQL language so that they can be performed programmatically on the populated ontology; and (iii) determining the expected answers to the CQs for the data in the ontology.

In a first step, we manually populated ImPO with data extracted from the files collected by the domain experts by randomly selecting four peptides and then manually verifying that the related data available were complete and spanned all the data files. This resulted in a total of 209 individuals characterized by 289 relations (i.e. object property assertions) and 399 attributes (i.e. data property assertions), with provenance from several studies (71–74). We highlight that even though the ontology was populated, data validation is beyond the scope of ImPO.

The process of formalizing the CQs into SPARQL followed the approach described by Potoniec *et al*. (75), beginning with identifying keywords in each CQ and gathering the corresponding vocabulary in the ontology to construct the query. For example, in the CQ ‘For each sample, extract the corresponding peptides’, we identified the keywords ‘sample’ and ‘peptides’, which have corresponding homonymous classes in the ontology. This is followed by finding the ontological path between the entities, which in this example would be: ‘sample’ > ‘mass spectrometry’ > ‘spectrum’ > ‘spectrum-peptide identification’ > ‘peptide’. The final step consists in translating the path into SPARQL, resulting in query 13 of Supplementary File 2.

To determine the expected answers to the CQs for the data in the ontology, we first determined in which files and columns the required entities for each query were and then traced the relations between the entities across the files, based on the shared or similar column names that enabled relating different files. All the relevant information (the required entities and the shared columns that allow mapping between files) was recorded in a spreadsheet with the expected result of each query. Finally, the expected result was manually compared with the result of the SPARQL query for each CQ to assess accuracy. The complete list of 15 CQs and respective queries can be found in Supplementary File 2.

### ImPO instancing with RDF mapping language

The integration of all instance data with ImPO was done at a later date using the RDF mapping language (RML) (76). RML defines specific mapping rules from various serializations and data formats to the RDF data model. This process began by defining the mapping rules for each TSV data file described in Table 2, mapping the dataset’s column names to the ontology’s entities and producing RML files for the mappings between those files. The RML files were subsequently processed by RMLStreamer (77) to convert those mapping rules to RDF format that can be integrated into the KG. The RML files that contain the mappings are available on ImPO’s GitHub repository.

## Results and discussion

### Conceptual modeling

The final ER model (Figure 4) includes 27 entities, 61 attributes and 32 relationships that describe the immunopeptidomics domain in depth. Some aspects of the domain were straightforward to model and suffered few or no changes as the model was refined, but most aspects proved challenging to model, requiring several iterations. The challenges were due to the complexity and specificity of the immunopeptidomics domain, its novelty and rapid evolution, and the need to ensure the traceability of the experimental information.

The latter often translated into a more complex model than would otherwise be necessary for concepts and relations that appear simple superficially. One example of this is the relation between peptide and protein: biologically one would expect a simple part-whole relation, but in reality, a peptide can be part of one or more proteins and with a given associated probability; thus, while they can be directly related, the relation must have many-to-many cardinality (whereas a simple part-whole relation would be many-to-one) and an attribute of its own. Another, more complex example of this is the relation between peptide and sample: in a simplistic approach, we could have treated peptide as a part of a sample, which is true from a biological perspective but would fail to capture that the peptide was identified as part of that sample from the spectrum of a given MS assay; thus, to enable traceability, we can only relate ‘peptide’ to ‘sample’ indirectly, through ‘mass spectrometry’ and ‘spectrum’.

The complexity and specificity of the domain are apparent in concepts such as the HLA, the class of proteins that present peptides on the surface of cells as part of the immune system and the very foundation of the immunopeptidomics domain. Immunopeptidomics studies focus not only on characterizing the peptides presented by the HLA but also on inferring on which of the six possible HLA alleles each peptide was presented, based on sequence motifs (65). Thus, in our model, HLA is associated with a ‘motif’ which is derived from several ‘peptides’ but also directly associated with ‘sample’, to mirror the experimental data (even though a connection to ‘sample’ could be made indirectly from the ‘peptide’ as detailed in the previous example).

The novelty and rapid evolution of immunopeptidomics are evidenced in the concept of ‘Epitope Contig’, an emerging concept recently introduced by the International Centre for Cancer Vaccine Science team in Bedran *et al*. (65) to mean genomic regions with ‘mutations that overlap with highly immune-visible regions’ also called ‘genomic immune clusters’. During the conceptual design of ImPO, the definition of this concept was iteratively refined to ‘A proposed region on the human genome surveyed by the immune system at a high frequency’.

### Semantic modeling

#### ImPO overview

The ImPO ontology comprises 48 classes, 36 object properties and 39 data properties that formally define the immunopeptidomics domain and structure experimental immunopeptidomics data, as summarized in Table 3.

**Table 3.** Descriptive statistics of the Immunopeptidomics Ontology

Axioms	828
Classes	48
Object properties	36
Data properties	39
Individuals	3
Annotation assertion	435

ImPO captures information in two subdomains: experimental and biological, as described in the Conceptual Modeling section of the Methodology. The experimental subdomain focuses on immunopeptidomics assays, such as MS. It includes details about samples, experimental parameters, results, and outputs. Samples are obtained as technical or biological replicates from fresh or frozen tissues taken from organs, primary cells or cultured cell lines. Each sample is associated with cancer, a group of diseases characterized by the uncontrolled cellular growth and, in some cases, spread to other organs due to mutations.

In the biological subdomain, ImPO describes the analytes identified by the assays. Analytes encompass genes, transcripts, proteins, genomic hotspots, HLAs, motifs, mutations, peptides, post-translational modifications, and the reference genome. Genes produce transcripts, which generate proteins that can be cleaved into peptides identified by MS.

Genomic hotspots are genomic regions frequently recognized by the immune system. These regions can encompass genes or peptides and are associated with specific cancers.

It is noteworthy to clarify that the main concern of ImPO is to model immunopeptidomics data to the extent necessary to interconnect the various dimensions of the data, enable traceability and inference and support cancer research. The concern was not providing a deep high-level organization because (i) it was not necessary since existing ontologies like the NCIt already cater to applications that require classification and more importantly (ii) the other goal is to integrate ImPO in the KATY KG, whereby its shallow high-level structure will be complemented by that of existing ontologies.

**Figure 3.**
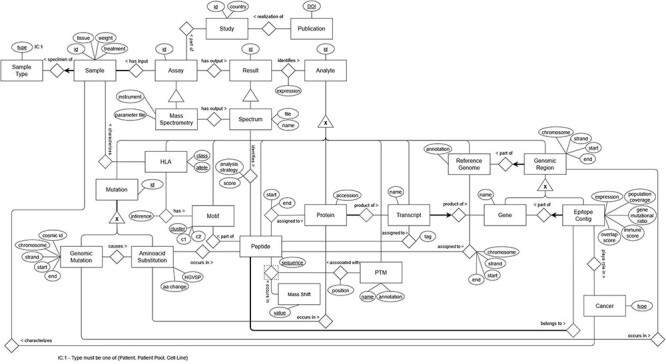
ER model representing the data from the immunopeptidomics domain.

**Figure 4.**
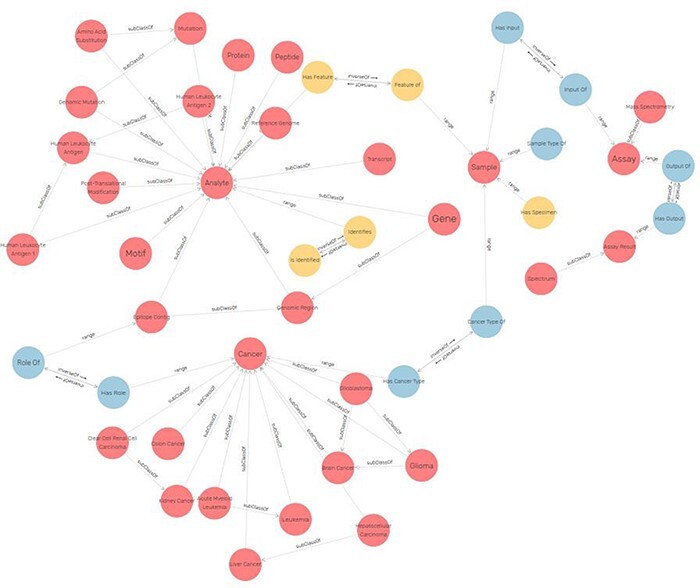
Excerpt of the ImPO visualized in GraphDB (78). The classes are represented as red circles and object property assertions between instances of the represented classes as directed arrows. Elements are labeled.

Figure 4 represents a subgraph of ImPO depicting some classes and object properties that are used to relate their individuals. There is a discrepancy between the number of entities present in the ER, 27, versus the 48 resulting classes in the ontology. This is due to the fact that some classes represent complex relations (with attributes) in the ER, such as ‘identifies’, ‘assigned to’ and ‘has’ (Figure 3). For instance, the relationship ‘assignment’ between a ‘peptide’ and a ‘protein’ in the ER with attributes ‘start’ and ‘end’ is represented by the class ‘Peptide-protein assignment’ in the ontology (Figure 4). Other additional classes were created as a result of the metamodeling of instances described before (e.g. cancer types).

To enable extensive cross-referencing with existing ontologies, we sought consistency with their modeling of core concepts, at the cost of making ImPO more complex. Next, we describe how ImPO represents a few relevant classes.

#### Representing genes in ImPO

**Figure 5.**
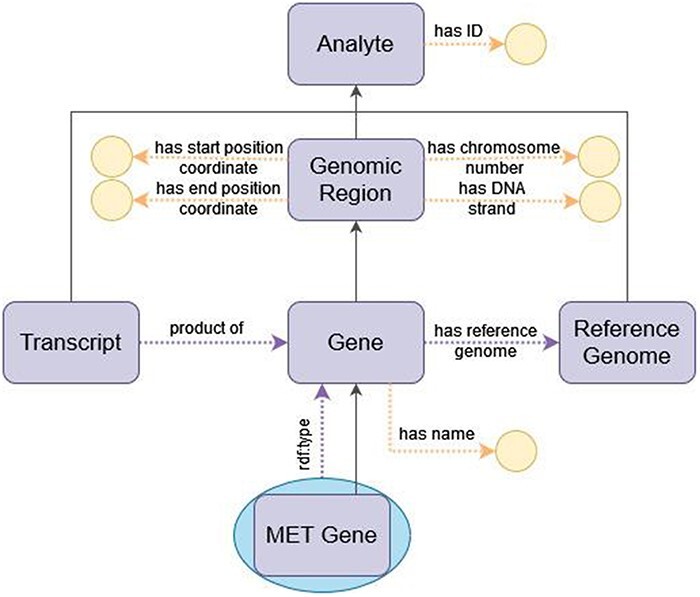
Diagram of the representation of a gene in ImPO. Purple rectangles represent classes, blue ellipses represent individuals, and yellow circles represent data values; black arrows represent subclass axioms; purple arrows represent rdf:type assertions of individuals or object property restrictions relating classes; yellow arrows represent data property restrictions of classes.

When representing genes, the divide between class and individual is less clear-cut than their definitions above may suggest and is entirely contingent on the application domain of the ontology. For example, in the NCIt (67), which is a catalog of cancer-related knowledge, human genes such as the MET gene are represented as subclasses of ‘Gene’. Under this view of the domain, the genes of each person are instances of the corresponding subclasses of ‘Gene’. However, in omics data, each person’s genes are represented through sets of mutations over the genes in the reference genome, so that there is only one instance of each gene (that of the reference genome). Thus, for an ontology that models omics data, it is more accurate and simpler to represent ‘MET gene’ as an individual of class ‘Gene’. OWL encompasses the possibility of declaring entities as both classes and individuals, known as metamodeling or punning, to accommodate the two viewpoints of an entity. ImPO accommodates both views by modeling genes as both individuals and classes. This metamodeling approach makes the ontology apparently more complex, but it is supported by OWL 2 DL and is inconsequential with respect to reasoning, as the two views of the concepts are treated as separate entities for that purpose. Figure 5 depicts a diagram of how a gene is represented in ImPO.

#### Representing cancer types in ImPO

Another example of modeling differences between existing ontologies and data pertained to the types of cancer (e.g. renal cancer), which are represented as subclasses of class ‘cancer’ in existing ontologies, but appear in the data as instances of ‘cancer’. In this case, we opted to conform only to the view of existing ontologies, rather than support the two, as it was deemed necessary to be able to distinguish the diseases of individual patients. This contrasts with the example of the genes because the latter represent the genes in the reference genome, and distinctions between the genes of individual patients or cancer tissues are made at the data level through the mutations over that reference, whereas there is no distinction at the data level between the cancers of individual patients. Thus, for cancer types, ImPO goes beyond the granularity of the data, and each patient’s cancer is modeled solely as an instance of the corresponding subclass of ‘cancer’.

#### Representing peptide identification through MS

**Figure 6.**
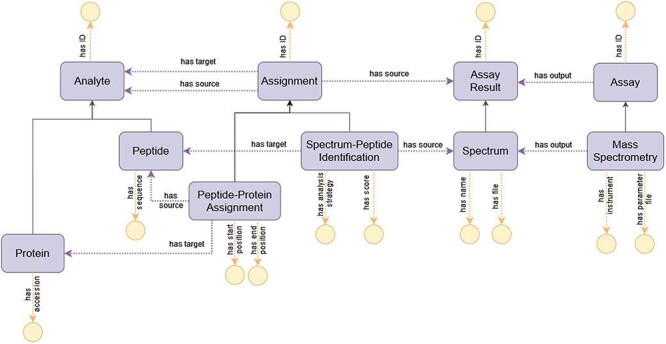
Diagram of the representation of the process of peptide identification through MS in ImPO. Purple rectangles represent classes, blue ellipses represent individuals, and yellow circles represent data values; black arrows represent subclass axioms; purple arrows represent rdf:type assertions of individuals or object property restrictions relating classes; yellow arrows represent data property restrictions of classes.

The modeling of the process of peptide identification through MS provides a comprehensive view of the upper-level structure of ImPO, as displayed in Figure 6: an ‘Assay’ such as ‘Mass Spectrometry’ is linked to an ‘Assay Result’ such as ‘Spectrum’ through the object property ‘has output’; an ‘Assignment’ represents the inferred relation between an ‘Assay Result’ and an ‘Analyte’ (e.g. ‘Spectrum’ to ‘Peptide’) or between two ‘Analytes’ (e.g. ‘Peptide’ to ‘Protein’) through the object properties ‘has source’ and ‘has target’ and enables the recording of attributes associated with the relation through data properties specific to the subclass of ‘Assignment’ (e.g. ‘has score’ in the case of ‘Spectrum-Peptide Identification’ or ‘has start position’ in the case of ‘Peptide-Protein Assignment’).

#### Representing epitope contigs

The epitope contig is a newly coined concept which means a continuous region within a gene that is most probable to undergo presentation on the surface of human cells by the HLA system. We develop such a gene-centric model to improve the interoperability of the data between two distinct communities in biomedical science—the proteomics and genomics communities. It is defined by one or more antigenic peptides (and characterized by their combined properties) found via immunopeptidomic analysis (be it from patient tissue or cell lines) that constitute a continuous region by overlapping in some portion and/or having their start and end positions next to each other.

The basis of acquiring data for this is to get sample tissue processed and analyzed via chromatography coupled with MS, producing chromatograms with embedded spectra. The MS data is then processed to peptides within the human proteome, as well as to find novel peptides not present in the reference human proteome (non-canonical peptides).

Because peptides are identified both within and outside the human reference proteome, it is convenient to annotate the regions of the genome these peptides may come from. This is accomplished by firstly, taking all the antigenic peptides, secondly, identifying their source proteins (it may be a one-to-many relation), then looking for the source transcripts, and, lastly, placing those transcripts on a region of a gene that is being transcribed. Taking into account a peptide’s position within a protein and possible modifications that the biological material can undergo throughout transcription and translation, we can provide a specific ‘address’ of a peptide within the genome (start, end, and chromosome). By looking at those properties, we can find aggregations or singular peptides that will constitute an epitope contig in genome coordinate space.

Each peptide included in an epitope contig can be characterized by its spectral count (information on how much of the MS data supported the presence of such peptide, equivalent to read depth in DNA/RNA sequencing) and associated HLA alleles. Those make a cumulative characterization of an epitope contig.

**Figure 7.**
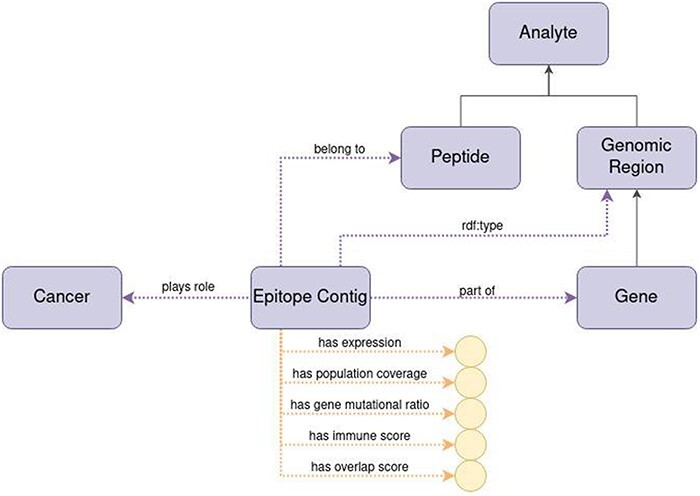
Diagram of the representation of the concept of epitope contig in ImPO. Purple rectangles represent classes, blue ellipses represent individuals, and yellow circles represent data values; black arrows represent subclass axioms; purple arrows represent rdf:type assertions of individuals or object property restrictions relating classes; yellow arrows represent data property restrictions of classes.

Figure 7 represents the epitope contig in ImPO with the following data properties:

- expression: the expression level of the associated gene
- population coverage: unique HLA alleles associated with included peptides
- immune score: a numerical indication whether given epitope contig can be treated as an immunogenic hotspot
- gene mutational ratio: frequency of mutations observed in that genomic region, based on all accumulated sequencing data
- overlap score: degree of overlap or similarity to other epitope contigs.

The epitope contig is also characterized by the specific chromosome number inside which the source nucleotides of all epitope contig’s peptides can be found, the start position (i.e. position of the first nucleotide of the first peptide in a gene) and the end position (i.e. the position of the last nucleotide of the last peptide in a gene).

#### Cross-referencing ImPO to existing ontologies

Table 4 presents the results of the alignment between ImPO and 30 biomedical ontologies identified as crucial to describe personalized oncology approaches (32). Using AgreementMakerLight’s (AML) automated matching approach to align both classes and properties, we found a total of 342 mappings to 31 ontologies.

**Table 4.** Results of the alignment of ImPO to the set of 34 ontologies identified as crucial to describe personalized oncology approaches, listing the number of mappings automatically generated by AML, the number of duplicate mappings that were removed, the number of incorrect mappings removed upon manual evaluation and the number of remaining mappings that were integrated into ImPO as cross-references

Acronym	Full name	AML mappings	Duplicates	Incorrect	Integrated
ACGT-MO (80)	ACGT Master Ontology	5	0	0	5
ATC (81)	Anatomical Therapeutic Chemical	2	0	0	2
CCTOO (82)	Cancer Care: Treatment Outcome Ontology	1	0	0	1
ChEBI (83)	Chemical Entities of Biological Interest	3	0	1	2
CL (84)	Cell Ontology	6	2	1	3
CLO (85)	Cell Line Ontology	18	14	0	4
CMO (86)	Clinical Measurement Ontology	0	0	0	0
DCM (87)	DICOM Controlled Terminology	0	0	0	0
DOID (88)	Human Disease Ontology	18	3	1	14
DTO (89)	Drug Target Ontology	15	14	0	1
EFO (90)	Experimental Factor Ontology	33	12	5	16
FMA (91)	Foundational Model of Anatomy	4	0	0	4
GENO (92)	Genotype Ontology	5	1	2	2
GO (12)	Gene Ontology	4	0	3	1
HCPCS (93)	Healthcare Common Procedure Coding System	1	1	0	0
HGNC (94)	HUGO Gene Nomenclature	0	0	0	0
HP (95)	Human Phenotype Ontology	16	6	0	10
ICDO (96)	International Classification of Diseases Ontology	1	1	0	0
IMGT (23)	Immunogenetics Ontology	4	0	0	4
LOINC (97)	Logical Observation Identifier Names and Codes	26	1	5	20
MONDO (98)	Mondo Disease Ontology	39	11	2	26
NCIt (67)	National Cancer Institute Thesaurus	47	0	3	43
OAE (99)	Ontology of Adverse Events	4	1	0	3
OMIM (100)	Online Mendelian Inheritance in Man	14	1	0	13
OBI (101)	Ontology of Biomedical Investigations	9	3	1	5
ONTIE (24)	Ontology for Immune Epitopes	4	4	0	0
OPMI (102)	Ontology of Precision Medicine and Investigation	5	4	1	0
ORDO (103)	Orphanet Rare Disease Ontology	8	0	3	5
PDQ (104)	Physician Data Query	15	1	1	13
PMAPP-PMO (105)	PMO Precision Medicine Ontology	19	0	0	19
PRO (21)	Protein Ontology	2	1	0	1
SO (106)	Sequence Ontology	10	0	1	9
	Total	342	86	31	225

**Figure 8.**
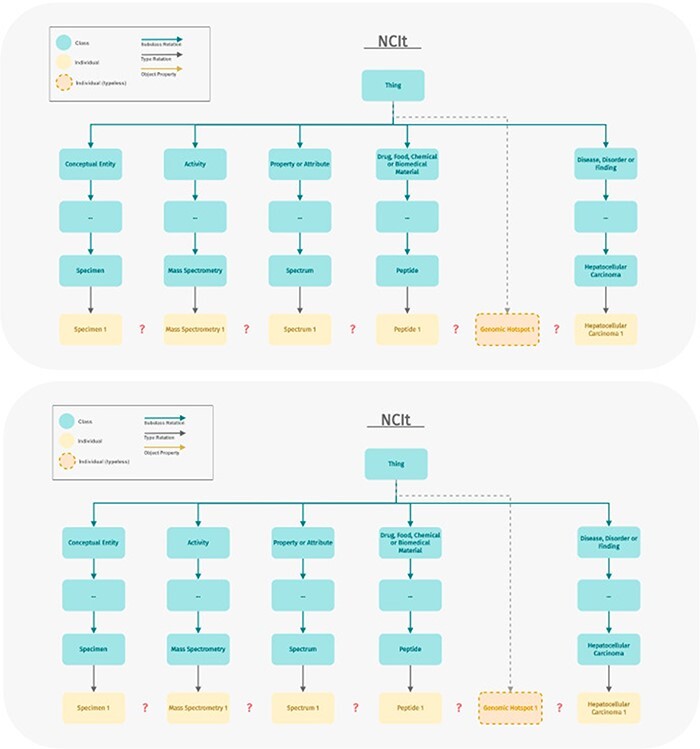
Example displaying the modeling difference between ImPO and NCIt. ImPO and NCIt model the biological entities relevant to immunopeptidomics in a strikingly different manner. ImPO and NCIt can both classify (most of) the data objects but only ImPO contains the properties and restrictions to interconnect them.

These mappings covered 38 out of 48 classes in ImPO. Not surprisingly, the ontology with the highest number of mappings was NCIt, a comprehensive ontology focused on cancer that follows a different semantic model. While many classes defined in ImPO also exist in NCIt, an ontology is more than just a hierarchy of classes. To effectively model immunopeptidomics data and the relationships between various data elements, appropriate object and data properties were required. Unfortunately, NCIt does not have suitable properties since it is primarily designed for classification and annotation rather than data modeling (Figure 7). Both ImPO and NCIt can classify most of the data objects, but only ImPO contains the necessary properties and restrictions to interconnect them. If we were to directly utilize NCIt, we would not only need to define additional properties but also impose semantic restrictions on some of its classes and even redefine some as individuals, which would be incorrect. Figure 8 demonstrates how NCIt is unable to provide information about the origin and provenance of specific genomic hotspots associated with particular types of cancer. Furthermore, NCIt lacks the capability to identify the peptides associated with these cancer types. In contrast, ImPO offers comprehensive traceability that addresses these limitations, providing researchers with valuable insights by allowing them to trace the detection of genomic hotspots and peptides back to their respective sample sources. This traceability provided by ImPO significantly enhances the understanding of the molecular characteristics and origins of various cancer types.

Due to the reuse strategy employed by many biomedical ontologies, the alignments included 86 duplicate mappings that connected an ImPO class to two occurrences of the same class in different ontologies. After removing these duplicates, the remaining mappings were manually validated and 226 were integrated into ImPO as cross-references.

### Evaluation

We evaluated ImPO by assessing its explanation consistency through reasoning, structural consistency through the identification of potential design pitfalls, and correctness and completeness by answering CQs.

#### Explanation consistency

The OWL reasoner HermiT was used to assess explanation consistency in ImPO in four runs. The first run revealed that ImPO was not OWL DL compliant due to a cardinality restriction on a transitive object property (‘part of’), and thus HermiT was unable to proceed. We solved this problem by creating a more specific object property to model the relation in question, instead of using the broad-scope ‘part of’ property. The second run of HermiT confirmed that ImPO was now OWL DL compliant and free of inconsistencies. The third run of HermiT was performed after we manually introduced data into the ontology for validation through CQs, to make sure that the data and ontology were consistent. This run revealed 62 inconsistencies pertaining to the data property assertions of individuals, which were incompatible with the domain or range restrictions of the respective data properties, due to errors in inserting the data. We corrected these errors, and the final run of HermiT confirmed that ImPO was consistent.

#### Structural consistency

OOPS was used to assess the structural consistency in two runs. The first run detected the following five pitfalls:

- P02. ‘Creating synonyms as classes’, caused by class ‘peptide genome assignment’ being erroneously declared as equivalent to ‘genomic region’, which happened due to lapse when editing the ontology in Protégé. It was addressed by removing the erroneous equivalent class declaration.
- P10. ‘Missing disjointness’, caused by no classes being declared as disjoint in ImPO. It was addressed by declaring disjointness between all classes that were conceptually disjoint.
- P11. ‘Missing domain or range in properties’, caused by property ‘part of’ lacking domain and range restrictions. It was not addressed as the property in question is broad scoped, and therefore, the lack of domain and range restrictions was intentional.
- P13. ‘Inverse relationships not explicitly declared’, caused by several object properties lacking an inverse property. It was partially addressed by declaring inverse properties for all object properties for which an inverse was plausibly useful to represent the data. The one exception was property ‘has reference genome’, which connects a gene to a reference genome, as the inverse declaration is never expected at the data level. It should be noted that declaring inverse properties, while generally a good practice, is in no way intrinsically necessary.
- P41. ‘No license declared’, caused by no license being declared in the ontology file, and addressed by declaring one by means of an ontology annotation.

After addressing the pitfalls as listed above, we ran OOPS a second time to ensure that no other pitfalls arose.

#### Correctness and completeness

The 15 SPARQL queries that encode the CQs were executed over ImPO populated with 209 individuals, and the results were compared with those manually determined from the data. If the ontology is correct and complete, then queries must retrieve all the entries manually determined and no others, meaning the queries must be 100% accurate. The results of this evaluation, listed in Table 5 for all CQs, validated that ImPO is correct and complete with respect to the application domain, as all queries had 100% accuracy. These results also highlight how semantic data modeling and integration facilitate data querying, as answering the CQs manually from the data required consulting and cross-referencing several data files.

**Table 5.** Results of the evaluation based on CQs, including the number of entries manually determined from the data for each question, the number of tuples found by the corresponding SPARQL query, the accuracy of the query and the number of data files consulted to answer each question

CQ	No. of entries found in raw files	No. of tuples by SPARQL query	Accuracy (%)	No. of files
1. For each sample, extract the corresponding peptides	20	20	100	1/10
2. For each HLA type, extract the corresponding peptides	4	4	100	3/10
3. For each epitope contig, extract the corresponding peptides	6	6	100	3/10
4. For each sample, extract the MS instrument	13	13	100	1/10
5. For each protein, extract the peptides	6	6	100	1/10
6. For each gene, extract the peptides	4	4	100	2/10
7. For each post-translational modification, extract the position in the corresponding peptide	3	3	100	1/10
8. Extract peptides that are shared across at least 10 non-disease-free samples	1	1	100	2/10
9. For each peptide, extract all associated mutations and their genomic coordinates	6	6	100	3/10
10. For each epitope contig, extract all associated mutations and their genomic coordinates	4	4	100	4/10
11. For each cancer, extract all associated mutations and proteins	4	4	100	4/10
12. For each cancer, extract all associated mutations and peptides	4	4	100	4/10
13. For each cancer, extract all associated mutations and PSMs	35	35	100	6/10
14. For each cancer, extract all associated mutations and PTMs	4	4	100	5/10
15. For each cancer, extract all associated mutations and epitope contigs	4	4	100	6/10

The purpose of the CQs was to evaluate the ontology’s completeness. The added value of ImPO will be fully realized by its full-fledged integration with other ontologies, which will open up numerous possibilities for future exploring and analyzing the data. This integration will enhance the capabilities of ImPO, enabling more extensive and interesting querying, such as ‘What are the pathways impacted by mutations in genomic hotspots?’, ‘How do mutations affecting specific pathways guide the selection of therapies for individual patients with specific types of cancer?’ and much more.

#### ImPO instancing

Here, we present the preliminary results of the instancing process of ImPO using RML (Supplementary Table S6). Our findings showcase the successful integration of a staggering 16 458 043 individuals into ImPO, representing 45 distinct classes. These results emphasize the potential of ontologies in effectively managing and organizing extensive datasets, demonstrating their crucial role in scientific research. Additionally, these results highlight the need for advanced technologies and tools like RML and RMLStreamer to aid in this effort, facilitating seamless data integration and enhancing research outcomes. The successful integration of such a large number of individuals into ImPO underscores the importance of employing robust frameworks and technologies to handle the ever-growing volume of scientific data.

## Conclusions

This work describes ImPO and its design process. ImPO aims to encapsulate and systematize the data generated by immunopeptidomics experimental processes and bioinformatics analysis. This systematization contributes to data integration and analysis, enabling future processing, inference and knowledge generation.

The design of ImPO followed a well-established methodology and best practices but also relied on strategic uses of ontology design patterns and OWL 2 features (such as metamodeling) to accurately depict the underlying domain and data. ImPO’s very specific scope and intended application to directly encode data motivated the decision of designing it as a standalone ontology, as existing ontologies that partially cover the domain are geared toward data annotation and classification and lack the necessary granularity for encoding data or concern themselves with other adjacent domains of narrow scope such as immunogenomics. We achieved interoperability between ImPO and 27 related ontologies or terminologies, including NCIt, Mondo Disease Ontology, Logical Observation Identifier Names and Codes and Experimental Factor Ontology, by incorporating mappings to those ontologies in ImPO, in the form of cross-references.

Although ImPO was designed based on expert knowledge to describe a specific data collection, this collection is large and representative, so ImPO can be easily employed to encode other datasets that cover the same domain. This malleability can be afforded through mapping languages, such as RML (76) that can be used to map between file column names and the ontology’s entities and then be automatically applied to generate instance data from data files. This will allow the construction of a KG for immunopeptidomics to bridge the gap between clinical proteomics and genomics and the integration with others (23) through extensive cross-references.

We expect ImPO to be a key piece in the coming-of-age of immunopeptidomics and in supporting a rich and standardized description of the large-scale data that emerging high-throughput technologies (2) will bring.

## Supplementary Material

baae014_Supp
