# Supplementary material for: The Immunopeptidomics Ontology (ImPO)
Source: Database (Oxford). 2024 Jun 10;2024:baae014. doi: 10.1093/database/baae014 (PMC11164101; doi:10.1093/database/baae014)
Supplement: baae014_Supp [file baae014_supp.zip › suppl_data/Supplemental Files.docx]

## Supplemental Materials

Supplemental File 1 - Immunopeptidomics search keywords used to query the datasets.

**Keywords:**

"Immunoprecipitation", "Immunopeptidome", "Peptidomics", "Affinity purification", "Mhc", "Peptidome", "Hla", "Immunopeptidomics", "Mhc class i", "Ip", "Hla peptidome", "Hla-b*27", "Hla class ii", "Neoantigens", "Immunoinformatics", "Hla-c", "Mhc class 1 ligands", "Proteogenomic cryptic mhc lc-msms maps", "Mhc class i antigen presentation pathway", "Mhc-i peptides", "Mhc I", "Immunopeptidome; hla; lc-ms/ms; netmhcpan; binding prediction", "Mhc ii", "Mhc-i peptide-loading complex", "Mhc affinity prediction", "Mhc-ii peptidomics", "Mhc ligandome", "Mhc i-associated peptides", "Mhc-i", "Mhc class ii", "Antigen presentation/ mhc class ii/ immunopeptidome/ peptide editing/ polymorphism", "Mhc-i peptidomics", "Shotgun proteomics; immunoprecipitation; meiosis; conserved proteins; meioc; ", "Anti-ha immunopurification", "Immunopeptidome; hla; lc-ms/ms; netmhcpan; binding prediction", "Personalized immunotherapy", "Immunoprecipation", "Immunoprecipiation", "Immunoaffinity purification", "Immunoprepicipitations", "Immunopurification", "Antigen presentation/ mhc class ii/ immunopeptidome/ peptide editing/ polymorphism", "Hla-ii", "Hla peptides", "Hla-e", "Hla-b*51", "Hla class i peptides", "Ducaf; hla-drb1*03:01", "Hla typing", "Hla-g", "Hla class I ligandome; hla class I peptide ligands; high ph reversed phase; strong cation exchange; pre-fractionation", "Hla-b40", "Hla binding motifs", "Hla-dm", "Hla-b27", "Immunopeptidome; hla; lc-ms/ms; netmhcpan; binding prediction", "Hla-b*58:01", "Hla-b*40:02 peptidome", "Hla-dr peptides", "Hla-dr", "Hla-a", "Hla-b57", "Hla class i", "Hla-i", "Hla-a2", "Hla-b", "Interferon gamma; proteomic analysis; hla class i; apm", "Hla-i peptides", "Hla-ligand", "Hla-b*57:03", "Hla-ligandomics", "Hla-a*29:02", "Hla-dr15", "Hla-class i", "Hla-restricted peptide"

**Instrument ids:**

"Q exactive", "Ltq orbitrap velos", "Orbitrap fusion lumos", "Ltq orbitrap elite", "Orbitrap fusion", "Orbitrap fusion etd", "Q exactive hf", "Q exactive plus", "Q exactive hf-x"

**Species ids:**

'Homo sapiens (human)'

**Period/year ids:**

‘2015’,’2016’,’2017,’2018’,‘2019’, ‘2020’, ‘2021’, ‘2022’.

### Supplemental File 2 - Competency questions and corresponding SPARQL-OWL queries.

1. For each sample extract the corresponding peptides.

PREFIX imnp: <https://raw.githubusercontent.com/liseda-lab/ImPO/main/ImPO.owl#>

SELECT DISTINCT ?sample_id ?peptide_sequence WHERE{

?sample imnp:has_id ?sample_id .

?mass_spec imnp:has_input ?sample .

?mass_spec imnp:has_output ?spectrum .

?spec_pep_id imnp:has_source ?spectrum .

?spec_pep_id imnp:has_target ?peptide .

?peptide imnp:has_sequence ?peptide_sequence .

}

2. For each HLA type extract the corresponding peptides.

PREFIX imnp: <https://raw.githubusercontent.com/liseda-lab/ImPO/main/ImPO.owl#>

SELECT DISTINCT ?hla_class ?peptide_sequence WHERE{

?hla imnp:has_class ?hla_class .

?motif_hla_assign imnp:has_target ?hla .

?motif_hla_assign imnp:has_source ?motif .

?motif imnp:has_part ?peptide .

?peptide imnp:has_sequence ?peptide_sequence .

}

3. For each Epitope Contig extract the corresponding peptides.

PREFIX imnp: <https://raw.githubusercontent.com/liseda-lab/ImPO/main/ImPO.owl#>

SELECT DISTINCT ?ec_id ?peptide_sequence WHERE{

?peptide imnp:has_sequence ?peptide_sequence .

?peptide imnp:part_of ?gh .

?gh imnp:has_id ?ec_id .

}

4. For each sample extract the MS instrument.

PREFIX imnp: <https://raw.githubusercontent.com/liseda-lab/ImPO/main/ImPO.owl#>

SELECT DISTINCT ?sample_id ?mass_spec_instrument WHERE{

?sample imnp:has_id ?sample_id .

?mass_spec imnp:has_input ?sample .

?mass_spec imnp:has_instrument ?mass_spec_instrument .

}

5. For each protein extract the peptides.

PREFIX imnp: <https://raw.githubusercontent.com/liseda-lab/ImPO/main/ImPO.owl#>

SELECT DISTINCT ?protein_id ?peptide_sequence WHERE{

?protein imnp:has_id ?protein_id .

?ppa imnp:has_target ?protein .

?ppa imnp:has_source ?peptide .

?peptide imnp:has_sequence ?peptide_sequence .

}

6. For each gene extract the peptides.

PREFIX imnp: <https://raw.githubusercontent.com/liseda-lab/ImPO/main/ImPO.owl#>

SELECT DISTINCT ?gene_id ?peptide_sequence WHERE{

?gene imnp:has_id ?gene_id .

?transcript imnp:product_of ?gene .

?protein imnp:product_of ?transcript .

?ppa imnp:has_target ?protein .

?peptide a imnp:peptide .

?ppa imnp:has_source ?peptide .

?peptide imnp:has_sequence ?peptide_sequence .

}

7. For each PTM extract the position in the corresponding peptide.

PREFIX imnp: <https://raw.githubusercontent.com/liseda-lab/ImPO/main/ImPO.owl#>

SELECT DISTINCT ?ptm_name ?awptm_position ?peptide_sequence WHERE{

?ptm imnp:has_name ?ptm_name .

?assoc_with_ptm imnp:has_target ?ptm .

?assoc_with_ptm imnp:has_source ?mass_shift .

?assoc_with_ptm imnp:has_position ?awptm_position .

?mass_shift imnp:occurs_in ?peptide .

?peptide imnp:has_sequence ?peptide_sequence .

}

8. Extract peptides that are shared across at least 10 non disease free samples.

PREFIX imnp: <https://raw.githubusercontent.com/liseda-lab/ImPO/main/ImPO.owl#>

SELECT DISTINCT ?peptide_sequence WHERE{

{

SELECT ?peptide_sequence ?sample WHERE {

FILTER EXISTS{?sample imnp:has_cancer_type ?cancer .}

{

SELECT ?peptide_sequence (count(distinct ?sample) as ?count_sample)

WHERE {

?sample a imnp:sample .

?mass_spec imnp:has_input ?sample .

?mass_spec imnp:has_output ?spectrum .

?spec_pep_id imnp:has_source ?spectrum .

?spec_pep_id imnp:has_target ?peptide .

?peptide imnp:has_sequence ?peptide_sequence .

}

GROUP BY ?peptide_sequence

HAVING (?count_sample >= 10)

}

}

}

}

9. For each peptide, extract all associated mutations and their genomic coordinates.

PREFIX imnp: <https://raw.githubusercontent.com/liseda-lab/ImPO/main/ImPO.owl#>

SELECT DISTINCT ?peptide_sequence ?geno_mut_id ?geno_mut_start_position

?geno_mut_end_position ?geno_mut_chromosome_number WHERE{

?peptide imnp:has_sequence ?peptide_sequence .

?aa_subst imnp:occurs_in ?peptide .

?aa_subst imnp:has_cause ?genomic_mut .

?genomic_mut imnp:has_cosmic_id ?geno_mut_id .

?genomic_mut imnp:has_start_position ?geno_mut_start_position .

?genomic_mut imnp:has_end_position ?geno_mut_end_position .

?genomic_mut imnp:has_chromosome_number ?geno_mut_chromosome_number .

}

10. For each Epitope Contig, extract all associated mutations and their genomic coordinates.

PREFIX imnp: <https://raw.githubusercontent.com/liseda-lab/ImPO/main/ImPO.owl#>

SELECT DISTINCT ?ec_id ?geno_mut_id ?geno_mut_start ?geno_mut_end ?geno_mut_chr

WHERE{

?gh a imnp:epitope_contig .

?gh imnp:has_id ?ec_id .

?genomic_mut imnp:occurs_in ?gh .

?genomic_mut imnp:has_cosmic_id ?geno_mut_id .

?genomic_mut imnp:has_start_position ?geno_mut_start .

?genomic_mut imnp:has_end_position ?geno_mut_end .

?genomic_mut imnp:has_chromosome_number ?geno_mut_chr .

}

11. For each cancer, extract all associated mutations and proteins.

PREFIX imnp: <https://raw.githubusercontent.com/liseda-lab/ImPO/main/ImPO.owl#>

PREFIX rdfs: <http://www.w3.org/2000/01/rdf-schema#>

PREFIX owl: <http://www.w3.org/2002/07/owl#>

SELECT DISTINCT ?cancer ?geno_mut_id ?has_prot_id WHERE{

?cancer_type a owl:Class .

?cancer_type rdfs:label ?cancer.

?cancer_ind a ?cancer_type .

?gh imnp:has_role ?cancer_ind .

?gh imnp:has_part ?genomic_mut .

?genomic_mut imnp:has_cosmic_id ?geno_mut_id .

?aa_subst imnp:has_cause ?genomic_mut .

?aa_subst imnp:occurs_in ?protein .

?protein imnp:has_id ?has_prot_id .

}

12. For each cancer, extract all associated mutations and peptides.

PREFIX imnp: <https://raw.githubusercontent.com/liseda-lab/ImPO/main/ImPO.owl#>

PREFIX owl: <http://www.w3.org/2002/07/owl#>

PREFIX rdfs: <http://www.w3.org/2000/01/rdf-schema#>

SELECT DISTINCT ?cancer ?geno_mut_id ?peptide_sequence WHERE{

?cancer_type a owl:Class .

?cancer_type rdfs:label ?cancer.

?cancer_ind a ?cancer_type .

?gh imnp:has_role ?cancer_ind .

?gh imnp:has_part ?genomic_mut .

?genomic_mut imnp:has_cosmic_id ?geno_mut_id .

?aa_subst imnp:has_cause ?genomic_mut .

?aa_subst imnp:occurs_in ?peptide .

?peptide imnp:has_sequence ?peptide_sequence .

}

13. For each cancer, extract all associated mutations and PSMs.

PREFIX imnp: <https://raw.githubusercontent.com/liseda-lab/ImPO/main/ImPO.owl#>

PREFIX owl: <http://www.w3.org/2002/07/owl#>

PREFIX rdfs: <http://www.w3.org/2000/01/rdf-schema#>

SELECT DISTINCT ?cancer ?geno_mut_id ?spectrum_id WHERE{

?genomic_mut imnp:has_cosmic_id ?geno_mut_id .

?aa_subst imnp:has_cause ?genomic_mut .

?aa_subst imnp:occurs_in ?peptide .

?spec_pep_id imnp:has_target ?peptide .

?spec_pep_id imnp:has_source ?spectrum .

?spectrum imnp:has_id ?spectrum_id .

?mass_spec imnp:has_output ?spectrum .

?mass_spec imnp:has_input ?sample .

?sample imnp:has_cancer_type ?cancer_ind .

?cancer_type a owl:Class .

?cancer_type rdfs:label ?cancer.

?cancer_ind a ?cancer_type .

}

14. For each cancer, extract all associated mutations and PTMs.

PREFIX imnp: <https://raw.githubusercontent.com/liseda-lab/ImPO/main/ImPO.owl#>

PREFIX owl: <http://www.w3.org/2002/07/owl#>

PREFIX rdfs: <http://www.w3.org/2000/01/rdf-schema#>

SELECT DISTINCT ?cancer ?geno_mut_id ?ptm_name WHERE{

?cancer_type a owl:Class .

?cancer_type rdfs:label ?cancer.

?cancer_ind a ?cancer_type .

?gh imnp:has_role ?cancer_ind .

?gh imnp:has_part ?genomic_mut .

?genomic_mut imnp:has_cosmic_id ?geno_mut_id .

?aa_subst imnp:has_cause ?genomic_mut .

?aa_subst imnp:occurs_in ?peptide .

?mass_shift imnp:occurs_in ?peptide .

?assoc_with_ptm imnp:has_source ?mass_shift .

?assoc_with_ptm imnp:has_target ?ptm .

?ptm imnp:has_name ?ptm_name .

}

15. For each cancer, extract all associated mutations and Epitope Contigs.

PREFIX imnp: <https://raw.githubusercontent.com/liseda-lab/ImPO/main/ImPO.owl#>

PREFIX owl: <http://www.w3.org/2002/07/owl#>

PREFIX rdfs: <http://www.w3.org/2000/01/rdf-schema#>

SELECT DISTINCT ?cancer ?geno_mut_id ?ec_id WHERE{

?cancer_type a owl:Class .

?cancer_type rdfs:label ?cancer.

?cancer_ind a ?cancer_type .

?gh imnp:has_role ?cancer_ind .

?gh imnp:has_part ?genomic_mut .

?gh imnp:has_id ?ec_id .

?genomic_mut imnp:has_cosmic_id ?geno_mut_id .

}
